# Correction to: TIGIT blockade enhances tumor response to radiotherapy via a CD103 + dendritic cell-dependent mechanism

**DOI:** 10.1007/s00262-023-03402-w

**Published:** 2023-02-21

**Authors:** Kaikai Zhao, Liyang Jiang, Youjiao Si, Shujie Zhou, Zhaoqin Huang, Xiangjiao Meng

**Affiliations:** 1 Department of Radiation Oncology, Shandong Cancer Hospital and Institute, Shandong First Medical University and Shandong Academy of Medical Sciences, Jinan, China; 2 Department of Radiology, Shandong Cancer Hospital and Institute, Shandong First Medical University and Shandong Academy of Medical Sciences, Jinan, China; 3 Cheeloo College of Medicine, Shandong University, Jinan, China; 4 Department of Radiology, Shandong Provincial Hospital Affiliated to Shandong First Medical University, Jinan, China; 5 Department of Radiation Oncology, Yantai Affiliated Hospital of Binzhou Medical University, Yantai, China; 6 Department of Radiology, Yantai Affiliated Hospital of Binzhou Medical University, Yantai, China

**Correction to: Cancer Immunology, Immunotherapy (2023) 72:193–209** 10.1007/s00262-022-03227-z

The original version of this article unfortunately contained a mistake. There were two mistakes in the Figs. 2 and 5.

In Fig. 2 amendments are:

Fig. 2a–b: Y-axis label,

Fig. 2a: Fig. 2a is wrong, it is the content of Fig. 2c, we modified this figure.

In Fig. 5 amendments are:

Fig. 5c: Statistical results are blocked.

The corrected Figs. 2 and 5 are given in the following page.

**Fig. 2:**
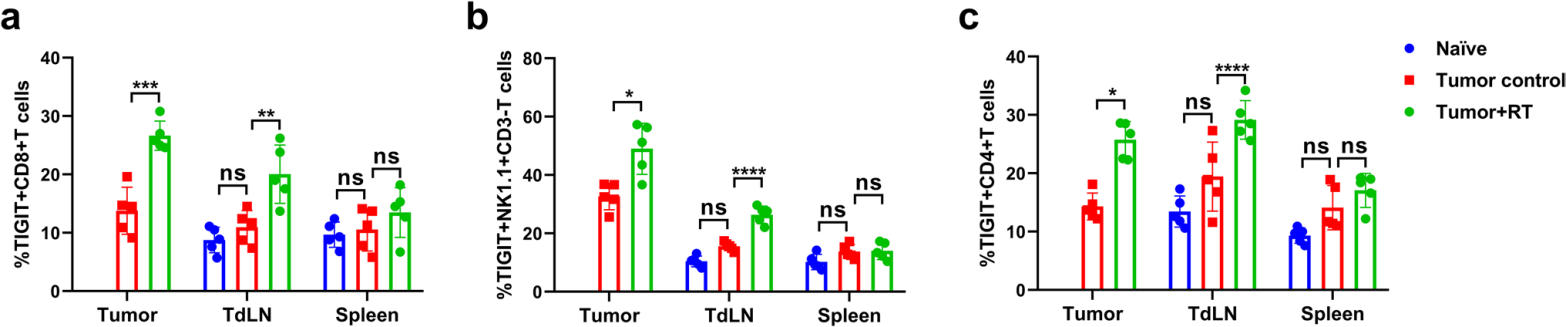
TIGIT expression was upregulated in tumor-infiltrating lymphocytes and TdLNs after RT in a mouse model. **a** CD8 + T cells had significantly higher expression of TIGIT in tumor and TdLN samples after RT. **b–c** CD4 + T-cells and NK cells also had increased TIGIT expression after RT in tumor and TdLN samples compared to those that did not receive RT. The spleen samples were unchanged. The gating strategy was shown in Fig. S2. *p < 0.05; ***p < 0.001; ****p < 0.0001. NS, not statistically significant; RT, radiotherapy; TdLNs, tumor draining lymph nodes; TIGIT, T cell immunoreceptor with immunoglobulin and ITIM (immunoreceptor tyrosine-based inhibitory motif) domains

**Fig. 5:**
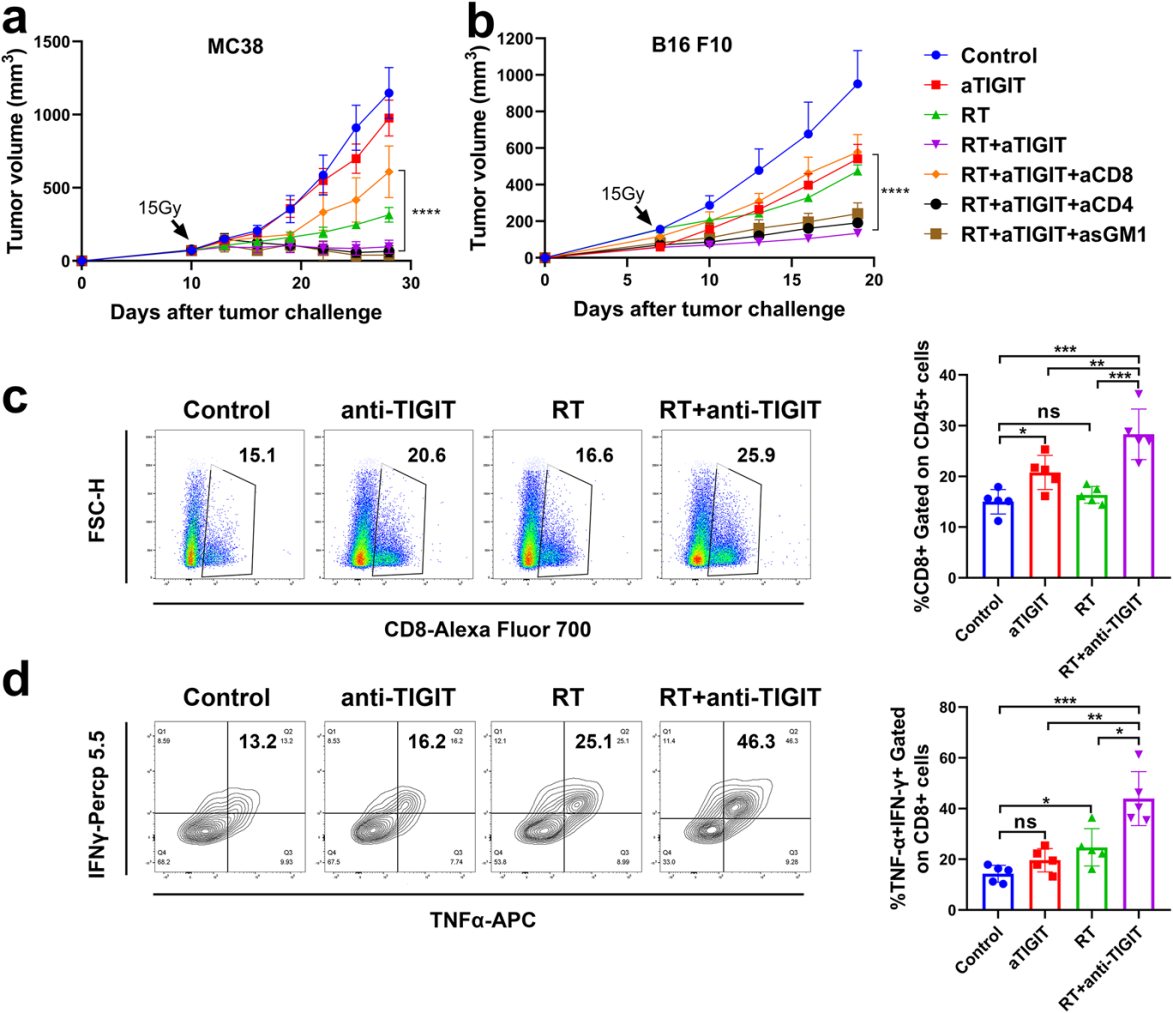
CD8 + T cells are required for effective RT and anti-TIGIT combination treatment. **a** Tumors (MC38) received 15 Gy and mice were treated with anti-TIGIT therapy, as described in Fig. 3a. Starting from 1 day before RT, 250 μg of depletion antibodies against CD8 + T, CD4 + T, and NK cells were injected intraperitoneally every 3 days for a total of four injections. **b** Tumors (B16 F10) received 15 Gy and mice were treated with anti-TIGIT therapy, as described in Fig. 3a. Depletion antibodies against CD8 + T cells, CD4 + T cells and NK cells were used. Representative data are shown from three **a** and two **b** experiments conducted with 5–6 mice per group. **c** Combination therapy greatly enhanced the antigen-specific response of CD8 + T cells. **d** Representative flow cytometry plots of IFN-*γ* and TNF-*α* expression on the CD8 + T cells extracted from the TILs of the control, anti-TIGIT alone, RT alone, and RT plus anti-TIGIT treatment groups (left). For intracellular cytokine staining, cells were stimulated with Cell Activation Cocktail (with Brefeldin A) (1:500) for 4–5 h before being harvested for cell surface staining, after which cells were fixed and permeabilized and stained with IFN-*γ* (interferon gamma) and TNF-*α* (tumor necrosis factor alpha). The gating strategy was shown in Fig S4. Quantification of IFN-γ/TNF-*α* dual production is shown on the right. Data are represented means ± SD (standard deviations) with two independent biological duplications. *p < 0.05; **p < 0.01; ***p < 0.001; ****p < 0.0001. NK, natural killer; NS, not statistically significant; RT, radiotherapy; TIGIT, T cell immunoreceptor with immunoglobulin and ITIM (immunoreceptor tyrosinebased inhibitory motif) domains; TILs, tumor infiltrating lymphocytes

